# Structural, magnetic, and magnetocaloric properties of R_2_NiMnO_6_ (R = Eu, Gd, Tb)

**DOI:** 10.1038/s41598-021-99755-2

**Published:** 2021-10-12

**Authors:** K. P. Shinde, E. J. Lee, M. Manawan, A. Lee, S.-Y. Park, Y. Jo, K. Ku, J. M. Kim, J. S. Park

**Affiliations:** 1 Department of Materials Science and Engineering, Hanbat National University, Daejeon, 34158 South Korea; 2 Fakultas Teknologi Pertahanan, Universitas Pertahanan Indonesia, Bogor, 16810 Indonesia; 3 Center for Scientific Instrumentation, Korea Basic Science Institute, Daejeon, 34133 South Korea

**Keywords:** Magnetic properties and materials, Ceramics

## Abstract

The crystal structure, cryogenic magnetic properties, and magnetocaloric performance of double perovskite Eu_2_NiMnO_6_ (ENMO), Gd_2_NiMnO_6_ (GNMO), and Tb_2_NiMnO_6_ (TNMO) ceramic powder samples synthesized by solid-state method have been investigated. X-ray diffraction structural investigation reveal that all compounds crystallize in the monoclinic structure with a P2_1_/n space group. A ferromagnetic to paramagnetic (FM-PM) second-order phase transition occurred in ENMO, GNMO, and TNMO at 143, 130, and 112 K, respectively. Maximum magnetic entropy changes and relative cooling power with a 5 T applied magnetic field are determined to be 3.2, 3.8, 3.5 J/kgK and 150, 182, 176 J/kg for the investigated samples, respectively. The change in structural, magnetic, and magnetocaloric effect attributed to the superexchange mechanism of Ni^2+^–O–Mn^3+^ and Ni^2+^–O–Mn^4+^. The various atomic sizes of Eu, Gd, and Tb affect the ratio of Mn^4+^/Mn^3+^, which is responsible for the considerable change in properties of double perovskite.

## Introduction

The worldwide need for cooling has increased because of global warming; cooling technology for food storage, safe pharmaceuticals, storage of hydrogen at cryogenic temperature, and protection of human being against excessive heat with high prices seeks cheaper and conservational cooling technology. Magnetic refrigeration technology, which work on the magnetocaloric effect (MCE) of magnetic materials, has attracted the interest of numerous research groups over traditional gas refrigeration. It has several advantages, including being highly energy efficient, environmentally friendly, and cost effective^1–4^. The MCE is a phenomenon of the coupling effect between the magnetic moment alignment with application and removal of magnetic field, which is strongly associated with the magnetic phase transition. It is also described as a variation of the isothermal magnetic entropy change or an adiabatic temperature change take place when the magnetic materials is kept under the changing magnetic field^5,6^. The scientific community interested in magnetic refrigerant materials with a significant MCE at ambient temperature as well as the cryogenic temperature region at lower magnetic field because of its applicability in magnetic refrigeration^7,8^. By using the conventional methods, oxides are synthesized on a large scale and different magnetic elements easily added as dopants. The transition temperature can be tuned by adjusting experimental conditions and doping elements. It has been reported that the oxides materials based on perovskite and double perovskite have been shown to improve magnetocaloric effect and relative cooling power (RCP)^9–12^.

Double perovskites (DPs) with the general formula *A*_*2*_*BB′O*_*6*_ have highly interesting chemical and physical properties with a wide variety of uses, which has attracted numerous research groups in the last decade. It is reported that, the atomic combination is very flexible with A, B, and B' elements are changed, resulting into the interesting property such as magnetoresistance^13,14^, ferroelectricity^15^, magneto-capacitance^16^, and magnetic refrigeration^17–19^. Jia et al*.* studied the magnetocaloric effect in Ln_2_(Ni/Co)MnO_6_ and discovered that these DPs exhibit multiple magnetic phase transitions, and Tc decreases with decreasing ionic radii of rare earth elements^20^. The magnetic transition from ferromagnetic to paramagnetic state in the double perovskite is related with the Ni^2+^/Co^2+^–O–Mn^4+^ superexchange mechanism. Balli et al. investigated the La_2_(Ni/Co)MnO_6_ compounds and discovered that the ordered phase of La_2_NiMnO_6_ single crystals has a high refrigerant capacity around 300 K when compared with high MCE materials^21,22^. Chakraborty et al*.* investigated and compared R_2_NiMnO_6_ (R = Pr, Nd, Tb, Ho and Y) with perovskite materials which are having comparable Curie temperature^23^. Su et al*.* conducted comparative research of Eu_2_NiMnO_6_ and Dy_2_NiMnO_6_ double perovskite and reported that – ΔS_M_ reaches to 4.0 J/kg K and 5.2 J/kg K with ΔH = 7 T^24^. The development of the Griffiths phase in Dy_2_NiMnO_6_ compound was seen due to spin-phonon coupling caused by the lower ionic radii of rare earth element (Ln^3+^). The essential factor in determining structural and magnetic or magnetocaloric properties in double perovskites is cation ordering between B and B' elements, as well as distortion in bonding owing to various A-site elements, which is thought to be the cause of complimentary findings. The small distortions caused by defects and disorders may cause redistribution of electron density, resulting in substantial changes in electronic characteristics, magnetic ordering, and magnetocaloric properties. Unfortunately, few research have been conducted to quantitatively demonstrate the relationship between the structure of double perovskite, namely B–O–B′ bonding, bond length, and superexchange mechanism, and subsequent magnetocaloric characteristics in these double perovskite systems.

In this article, we studied the influence of rare earth element ionic size in R_2_NiMnO_6_ (R = Eu, Gd, Tb) double perovskite on structural, magnetic, and magnetocaloric properties at cryogenic temperatures. The various sizes of rare earth elements influence the bond angle studied by Rietveld refinement and X-ray photoemission (XPS) revealing the Mn^4+^/Mn^3+^ ionic distribution in the double perovskite. At cryogenic temperatures, the magnetocaloric characteristics are revealed to comprehend its usage as a magnetic refrigerant for cooling applications.

## Results and discussion

**Figure 1:**
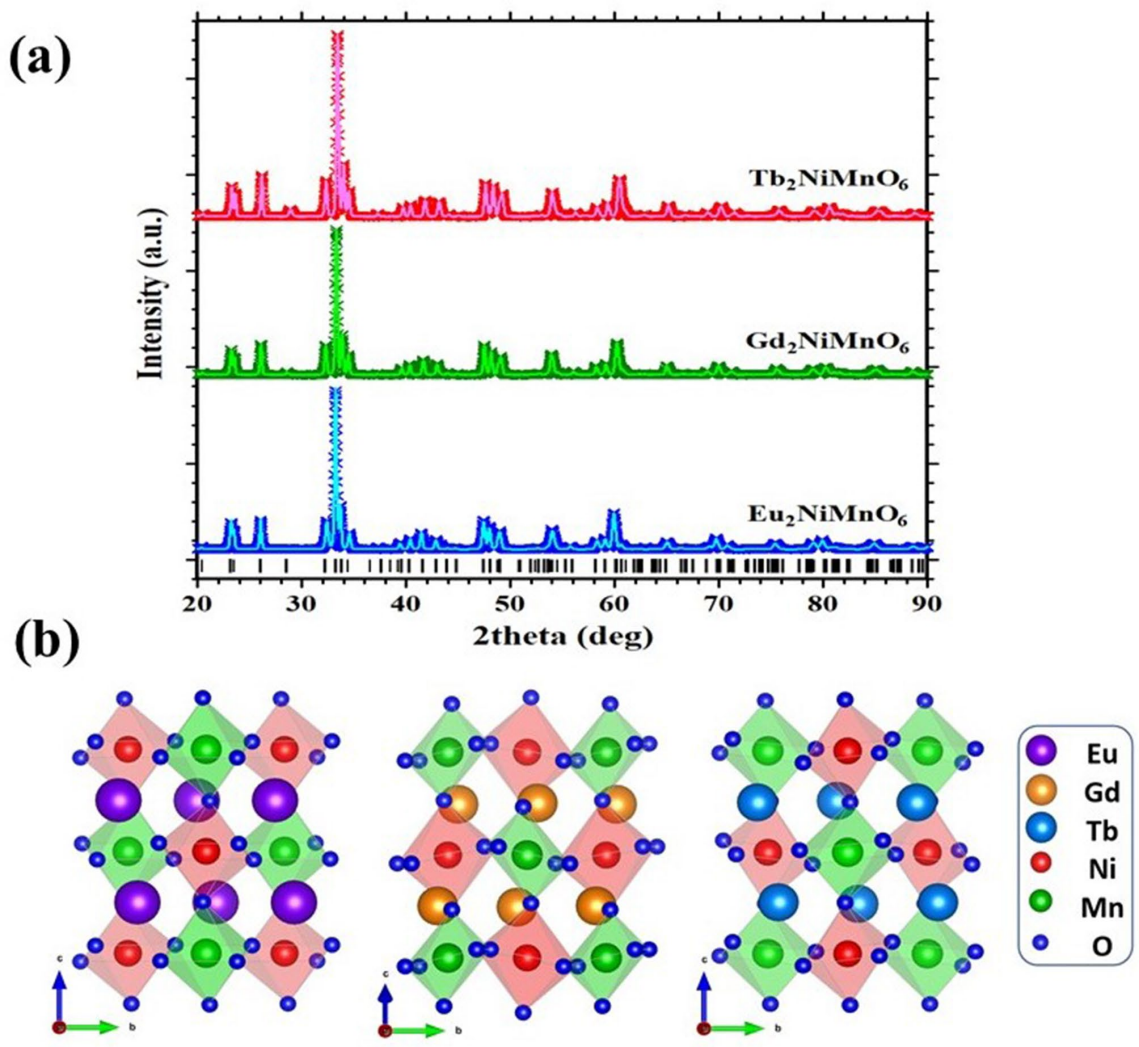
(**a**) Rietveld refinement of x-ray diffraction data. (**b**) Monoclinic crystal structure of ENMO, GNMO, and TNMO samples. In crystallographic structure blue, green, olive, pink, gray, and red spheres represent Eu^3+^, Gd^3+^, Tb^3+^, Ni^2+^, Mn^4+^, and O^2-^ ions, respectively.

The rietveld refinement of room temperature X-ray diffraction data is used to study the phase purity and crystal structure of the samples. The rietveld refined XRD pattern of the ENMO, GNMO, and TNMO samples are shown in Fig. 1a. According to the diffraction patterns, all the investigated compositions crystallized in the monoclinic crystal system with space group P2_1_/n. The structural parameters are refined using the TOPAS program by Rietveld's profile fitting technique^25^. Table 1 show the refinement parameters of the investigated samples, such as lattice parameters, bond length, goodness of fit (χ^2^), and bond angle. It is observed that when the atomic number of rare earth elements increases, cell volume and crystal density increase, but cell volume declines. The atomic arrangement in monoclinic structure of ENMO, GNMO, and TNMO are shown in Fig. 1b. When compared to ENMO and TNMO, the crystal structure of GNMO is significantly tilted (β = 124°). The octahedral arrangement of NiO_6_ and MnO_6_ edges are shared to create a cross chain network in a single unit cell. The tilting of angle of the octahedron and the stability of the perovskite changes with the growth of the radius of the A, B, and B′ metal ions in the double perovskite structure^26^. The structurally stability of double perovskite, is defined by Goldschmidt tolerance factor (*t*) which is given by relation,

1 $$t = \frac{{r_{A} + {\text{ro}}}}{{\sqrt {2\left[ {ro + \left( {\frac{{r_{B} + r_{{B^{\prime}}} }}{2}} \right)} \right]} }}$$

where, r_A_, r_B_, r_B′_, and r_O_ represent ionic radii of A, B, B'_′_ and O site element in double perovskite.

The *t* values of ENMO,GNMO, and TNMO determined by using above Eq. (1) are 1.02, 1.01, and 1.00, respectively. As we know, the double perovskite structure is stable when the value of *t* is between 0.78 and 1.05. The calculated values of *t* are within this range, indicating that the investigated samples are stable^3^.

**Table 1:** Structural parameters for ENMO, GNMO, and TNMO from the Rietveld refinement.

	Eu_2_NiMnO_6_	Gd_2_NiMnO_6_	Tb_2_NiMnO_6_
Space group	*P2*_*1*_*/n*	*P2*_*1*_*/n*	*P2*_*1*_*/n*
Cell mass (g/mol)	1027.11	1048.26	1054.96
Cell volume (Å^3^)	222.86	221.56	219.58
Crystal density (g/cm^3^)	7.653	7.857	7.978
**Lattice parameters**			
*a* (Å)	5.3206	5.2915	5.2706
*b* (Å)	5.5251	5.4103	5.5358
*c* (Å)	7.5811	9.2220	7.5261
*β* (°)	90.003	124.976	90.158
Rexp	4.97	4.43	4.30
Rwp	9.40	6.25	8.05
GoF	1.89	1.41	1.87
**Bond length (Å)**			
Ni–O	1.9961	2.0781	1.8256
Mn–O	1.9481	1.9120	2.0444
**Bond angle (°)**			
Ni–O–Mn	161.84	153.07	147.30

**Figure 2:**
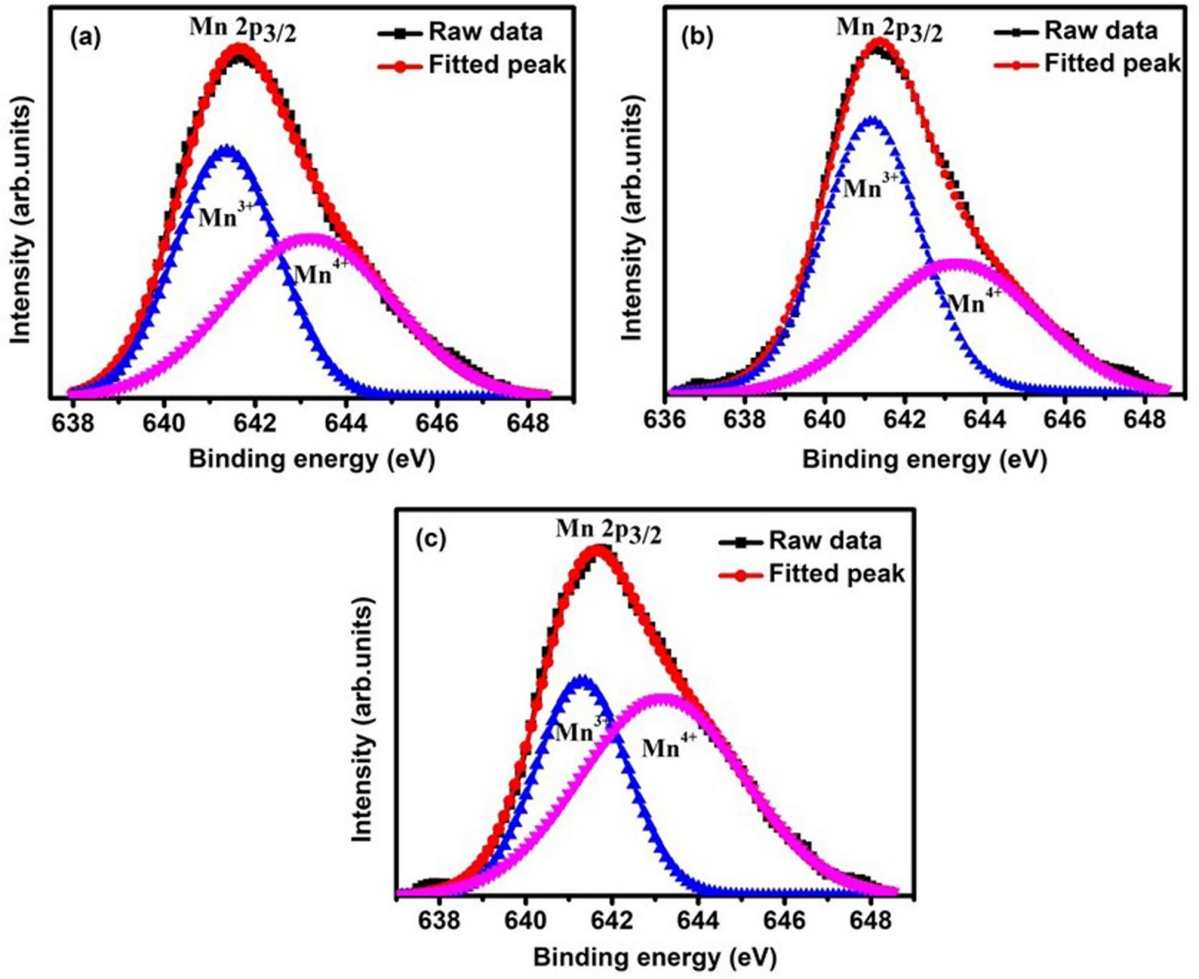
High-resolution XPS spectra of Mn2p for (**a**) ENMO, (**b**) GNMO, (**c**) TNMO samples.

The X-ray photoemission spectroscopy (XPS) technique is used to investigate the chemical oxidation states and the ligand coordination of the samples. The oxidation state analysis of Manganese (Mn) in ENMO, GNMO, and TNMO samples were done, and XPS spectra for Mn2p are shown in Fig. 2a–c. To fit the spectra, Shirley background subtraction was employed. The deconvoluted XPS peak of Mn 2*p*^3/2^ breaks into two peaks at 641.2 eV and 643.2 eV, which correspond to Mn^4+^ and Mn^3+^, respectively^27^. The ratio of Mn^4+^/Mn^3+^ for ENMO, GNMO, and TNMO were found to be 1.02, 0.76, and 1.64, respectively, showing that Mn^4+^ is more dominant in ENMO and TNMO, whereas Mn^3+^ is more dominant in GNMO. The Mn^4+^/Mn^3+^ ratio demonstrates the change in surface oxidation state induced by distinct A-site rare earth element with varying ionic radii and which indicates that the superexchange mechanism of Ni^2+^–O–Mn^3+^ and Ni^2+^–O–Mn^4+^ in the investigated compounds are distinct. However, the structural instability caused by the various ionic sizes of the A-site elements in double perovskite causes uncertainty in the Mn^4+^/Mn^3+^ ratio, which influences the magnetic properties of the compounds.

**Figure 3:**
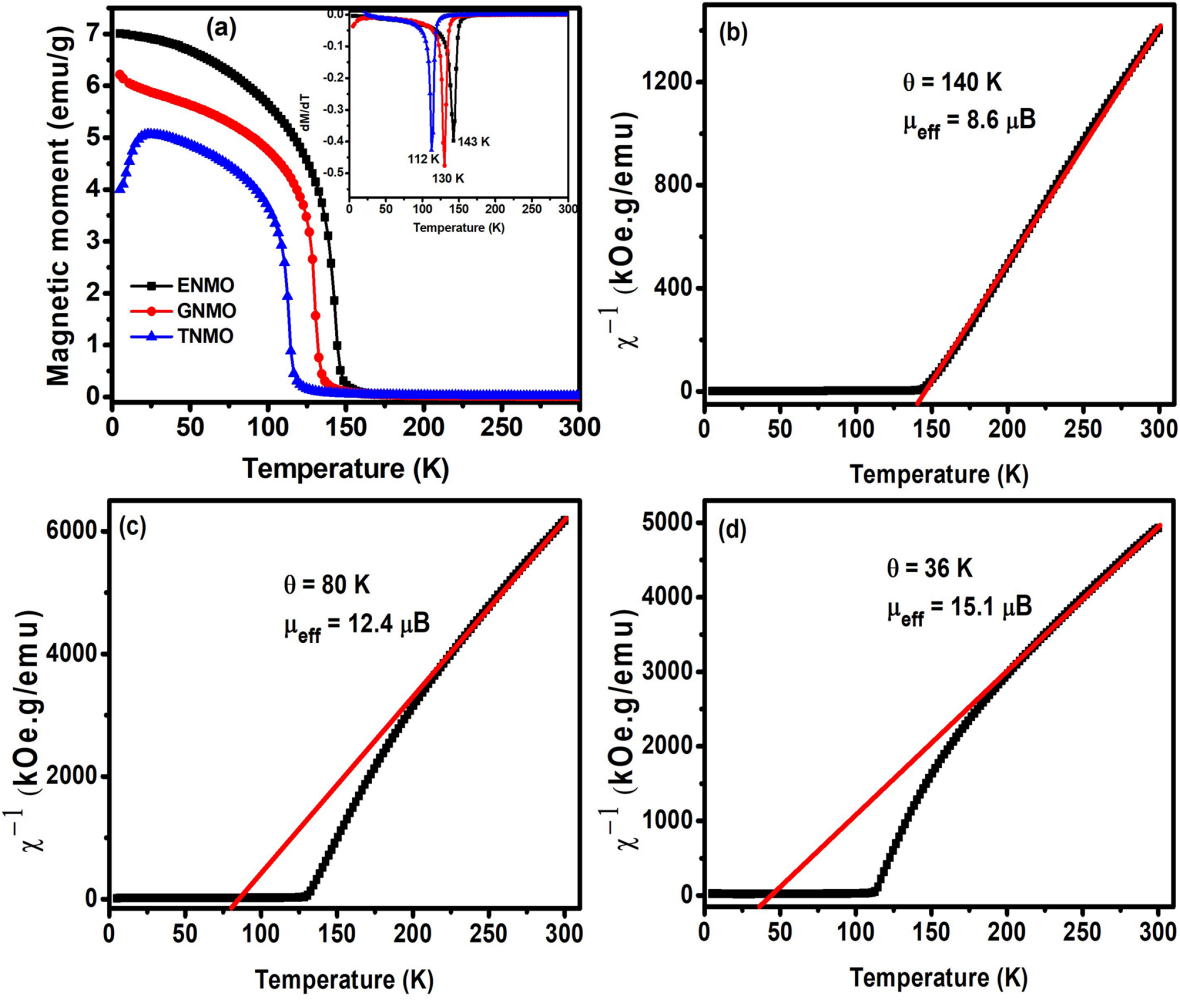
(**a**) The temperature dependent magnetization under the magnetic field of 100 Oe. Inset show variation of dM/dT curve with temperature. (**b**–**d**) Temperature dependence of the inverse magnetic susceptibility and Curie–Weiss fitting for ENMO, GNMO and TNMO samples.

Figure 3a depicts temperature-dependent magnetization (MT) curves of ENMO, GNMO, and TNMO samples recorded with a 100 Oe applied magnetic field between 2 and 300 K. It is apparent that when the temperature increased, the magnetization in the samples dropped due to the magnetic phase transition temperature from ferromagnetic to paramagnetic. The MT curve verifies the ferromagnetic to paramagnetic phase change caused by the well-known superexchange exchange phenomenon linked with Ni^2+^–O–Mn^4+^/Ni^2+^–O–Mn^3+^. To calculate the Curie temperature (T_C_), the temperature dependences of dM/dT for all samples are presented in the inset of figure. It is defined as the minimum of the dM/dT curve, and the Curie temperatures for ENMO, GNMO, and TNMO samples are found to be 143, 130, and 112 K, respectively. The Curie temperature progressively moves to lower temperature when the rare earth size Eu^3+^ (0.947 Å) > Gd^3+^ (0.938 Å) > Tb^3+^ (0.923 Å) declines, which is likely related to reductions in the Ni–O–Mn bond angle [161.84 (Eu), 153.07 (Gd), and 147.30 (Tb)]^28^. However, as the size of rare earth ions decreases in double perovskite R_2_CoMnO_6_ (R = La,…,Lu) compounds, the transition temperature changes linearly from 204 K for La_2_CoMnO_6_ to 48 K for Lu_2_CoMnO_6_ due to long-range magnetic order originating from the dominating Co^2+^ and Mn^4+^ superexchange interactions^29,30^. The ordered alignment of the Ni^2+^–O–Mn^4+^ superexchange interaction to the ferromagnetic interaction found in double perovskite compounds following the Goodenough–Kanamori rules between empty and half-filled orbitals of transition metals. Due to partial disorder of Ni and Mn, Ni^2+^–O–Ni^2+^ and Mn^4+^–O–Mn^4+^ display mild antiferromagnetic interactions^31^. In the case of TNMO, it is noted that magnetization decreases at low temperatures (2–25 K), similar with Dy_2_NiMnO_6_, and this is caused by anti-parallel alignment of rare earth (Tb) magnetic moments as opposed to transition metal (Ni and Mn) magnetic moments. The temperature dependent inverse susceptibility χ^−1^(T) is shown in Fig. 3b,c,d. In paramagnetic region, the linear fitting observed for the experimental χ^-1^(T) curve with the Curie–Weiss (C–W) equation, χ = C/(T − θ), where C is the Curie constant, and θ corresponds to paramagnetic Curie–Weiss temperature. The values of Curie–Weiss temperature (θ) were discovered to be 140 K, 80 K, and 36 K, respectively, and the computed values of effective magnetic moments are 8.6, 12.4, and 15.1 µB/f.u. for ENMO, GNMO, and TNMO, which are close to the corresponding theoretical values of 7.0, 12.2, and 14.6 µB/f.u. The discrepancy between the theoretical and calculated effective magnetic moments is related to the extent of Mn^3+^ and Mn^4+^ in the sample. The positive Curie–Weiss temperatures for the investigated DPs confirm the ferromagnetic phase transition occurs. The Curie–Weiss fitting demonstrates that the Griffith phase evolves with decreased ionic size of Eu, Gd, and Tb in double perovskite. Griffith phase is mostly found in manganites, although cationic disorder caused by mixed occupancy at the B-site element (Ni) and the smaller size of the A-site element (Eu, Gd, and Tb) creates a similar effect in double perovskite structure. It is seen from the Fig. 3b–d that with decrease in temperature from 300 K, χ^−1^ obeys Curie Weiss law until 200 K, with further decrease in temperature a downturn is evident in case of GNMO and TNMO sample in χ^−1^ vs T plot which is a typical Griffith Phase feature. Bhatti et al. found reported similar Griffith phase behavior in nanocrystalline Gd_2_CoMnO_6_. The deviation from the Curie–Weiss law fitting of χ^−1^ increases with magnetic disorder, which is caused by the various ionic sizes of rare earth elements in current double perovskites^32^.

**Figure 4:**
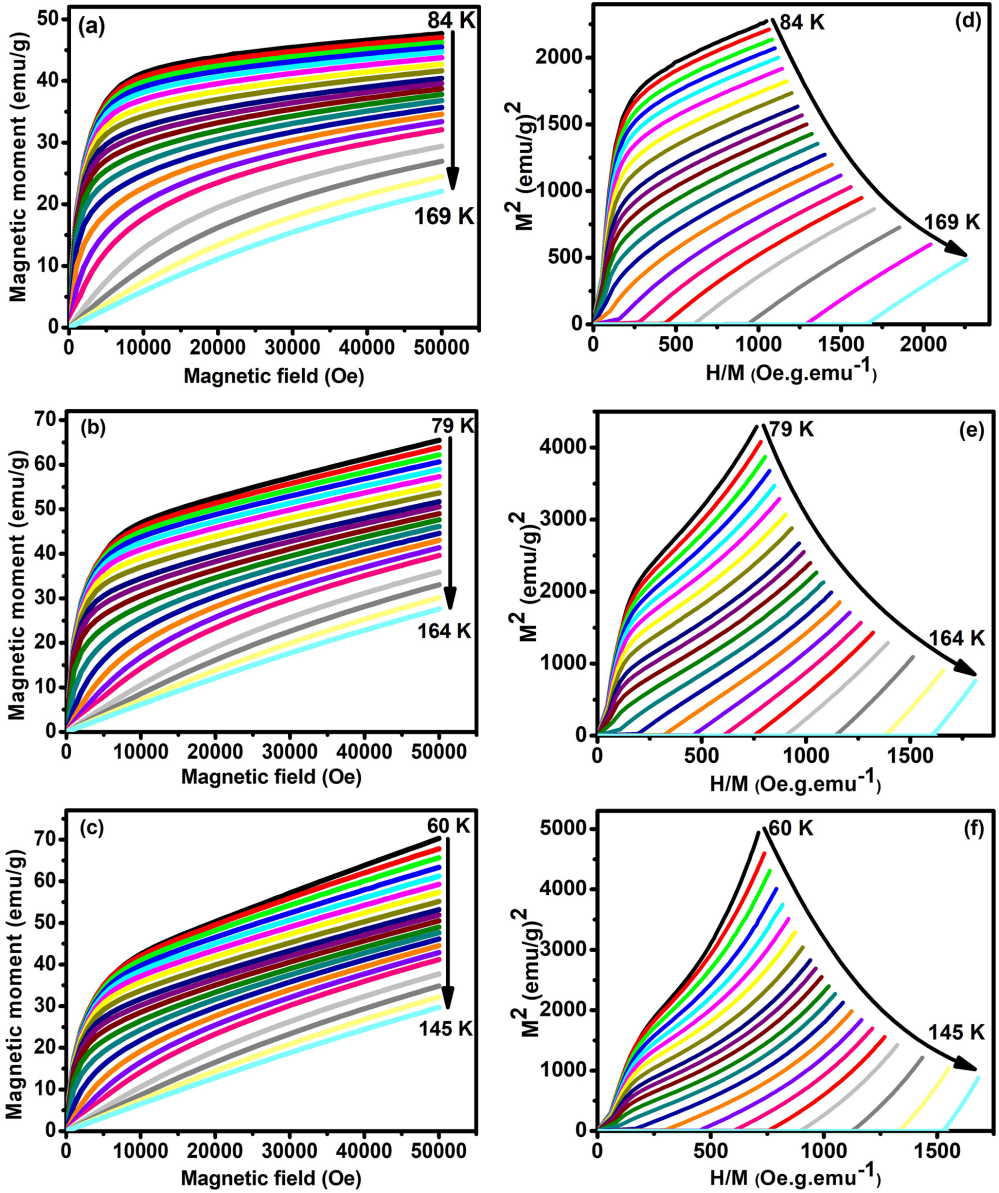
(**a**–**c**) Isothermal magnetization curves (MH) around T_C_ (**d**–**f**) Arrott plot for ENMO, GNMO and TNMO samples. The positive slope of Arrott plots confirms second-order magnetic phase transition in studied samples.

To examine the magnetocaloric properties, the isothermal magnetization (MH) curves at various temperatures were measured before and after the T_C_. The temperature intervals ΔT = 3 K near T_C_ and ΔT = 5 K in the rest of temperature region were kept constant. Figure 4a–c depicts isothermal magnetization of ENMO, GNMO, and TNMO samples in a magnetic field range of 0–5 T. The MH curves show that when the magnetic field is low, the MH curves increase quickly, and when the magnetic field is high, the MH curves strive to saturate, and this phenomenon is related with the ferromagnetic behavior of magnetic materials. The MH curves exhibit linear behavior at higher temperatures, confirming the paramagnetic nature of the materials, and this is owing to thermal agitation, which disorients the magnetic moments at higher temperatures. To understand, the order of magnetic phase transition, well-known Arrott plots (M^2^ vs H/M) were studied, and which are derived from the magnetic isotherms shown in Fig. 4d–f. According to Banerjee's criteria, the slope of Arrott plots is significant in determining the type of magnetic phase transition. The negative slope represents the first-order phase transition while the positive slope verifies the second-order phase transition^33^. The Arrott plot show a positive slope at all temperatures for the samples examined. As a result, we can confirm that the ferromagnetic–paramagnetic transition is of the second-order type. The “S”-shaped Arrott plot, indicates that GNMO and TNMO samples suffer a weak first-order phase transition, but ENMO totally exhibits second-order phase transition. The Arrott plot analysis indicates that the first order spin re-orientation at lower temperature resulted in an FM/AFM transition in these samples. The existence of disordered B-sites with magnetic ions of Ni and Mn in mixed valence states might explain the complex magnetic structure found in these compounds. In the case of a second order phase transition, the order of degree of magnetic domains, variation in lattice volume, and latent heat of phase transformation are all extremely modest. It is possible that it may be one of the reasons why magnetic entropy changes in second-order phase transition materials are lower than those in first-order phase transition materials.

The magnetic entropy change calculated by utilizing the isothermal magnetization data, shown in Fig. 4, and which is initiated by the changing the applied magnetic field from 0 to H is determined by applying the well-known Maxwell thermodynamic correlation, which is given by the equation^34^,

2 $$\Delta {S}_{M }\left(T, H\right)= {\int }_{0}^{H}{\left(\frac{\partial M}{\partial T}\right)}_{H} dH$$

where, ΔS is the magnetic entropy change, dH is the change in the applied magnetic field, M is the magnetization and T is the temperature.

**Figure 5:**
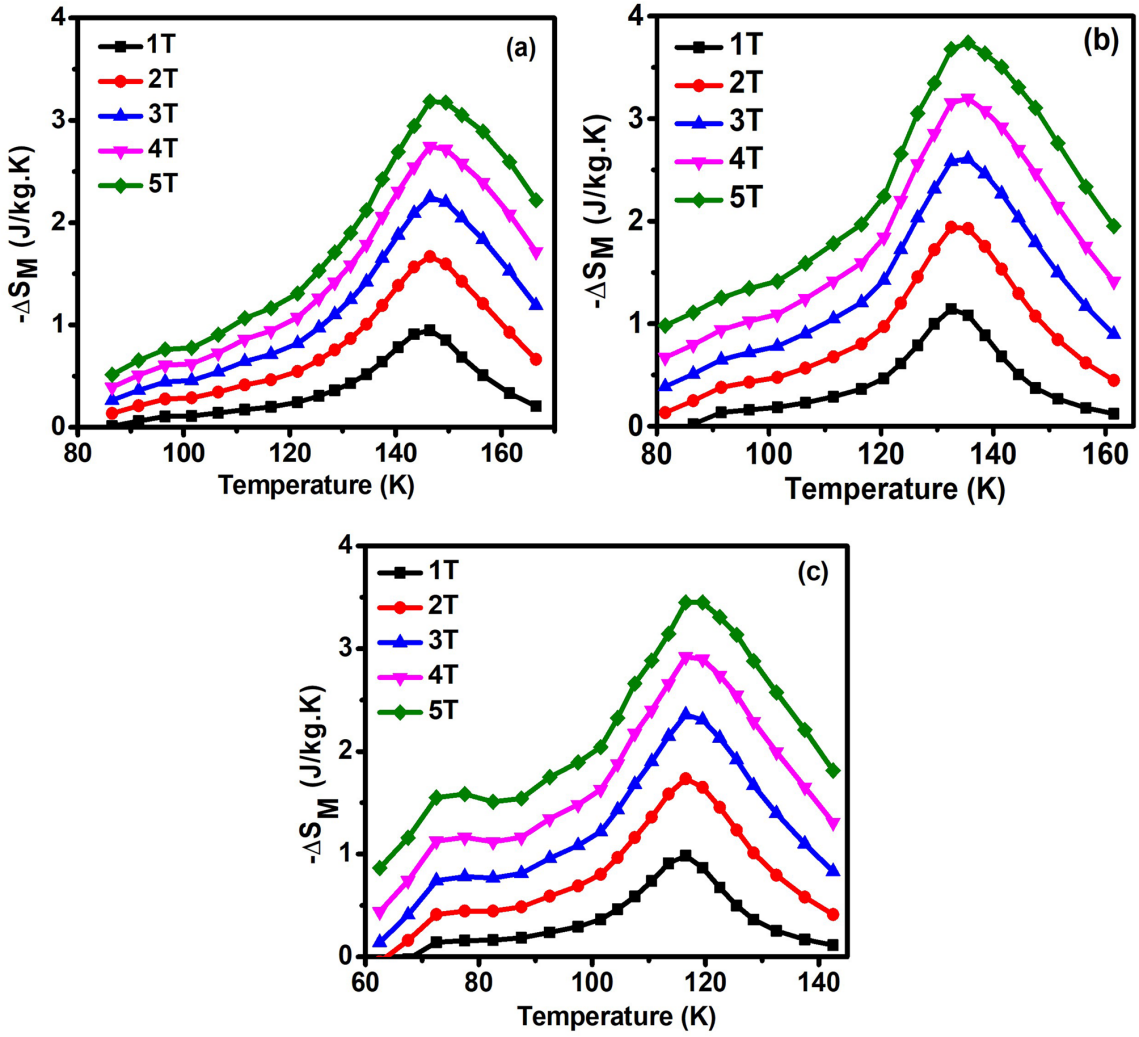
Magnetic entropy change (– ΔS_M_) with respect of temperature in the magnetic field 1–5 T for (**a**) ENMO, (**b**) GNMO and (**c**) TNMO samples.

The temperature dependence of magnetic entropy curves (− ΔS_M_) for ENMO, GNMO, and TNMO is shown in Fig. 5a–c for varied applied field from 0 to 5 T. All these samples exhibit a similar pattern, the – ΔS_M_ value achieves a maximum at the Curie temperature (T_C_) and increases with increasing H, possibly due to improved FM interactions. The maximum value of – ΔS_M_ is found to be around the magnetic phase transition temperature. Because of the deformed structure of the MnO_6_ and NiO_6_ octahedrons, the magnetic entropy change for TNMO sample exhibits a shoulder peak at lower temperature region and high magnetic field, which is attributed to the presence of antiferromagnetic exchange interaction such as of Ni^2+^–O–Ni^2+^ and Mn^3+^–O–Mn^3+^. The calculated – ΔS_M_ values are 3.2, 3.8, and 3.5 J/kgK at ΔH = 5 T for ENMO, GNMO, and TNMO samples, respectively. Rawat et al*.* reported the MCE characteristics of nanocrystalline Pr_2_CoMnO_6_ DPs produced by sol–gel with an average particle size of 192 nm, which were determined to be – ΔS_M_ = 1.98 J/kgK at a field change of 5 T and relative cooling power (RCP) of 110 J/kg^35^. Su et al*.* investigated the MCE characteristics in Eu_2_NiMnO_6_ and Dy_2_NiMnO_6_ and discovered that the highest value of magnetic entropy change – ΔS_M_ approaches 4.0 J/kg K and 5.2 J/kg K, respectively, for field changes of 0–7 T^24^. With, ΔH = 5 T, Chakraborty et al. found an – ΔS_M_ value of 6.2 J/kg K for Ho_2_NiMnO_6_ and 4.1 J/kg K for Tb_2_NiMnO_6_ double perovskite^23^. As compared with literature, this study discovered that when the ionic radii of rare earth elements reduce in double perovskite, the Griffith phase emerges. The effect of Griffith phase on magnetocaloric properties has not yet been reported. Set of experiments needed with different ionic radius of A-site and B-site element is under consideration.

Another essential measure for determining the efficacy of MCE materials is evaluating the cooling efficiency of the materials, which is referred to as relative cooling power (RCP). It is defined as, an amount of heat transferred between temperatures corresponding to the full width at half maximum of magnetic entropy change curve, and it is evaluated by the following equation,

3 $$RCP={- \Delta S}_{M }X {\delta T}_{FWHM}$$

**Figure 6:**
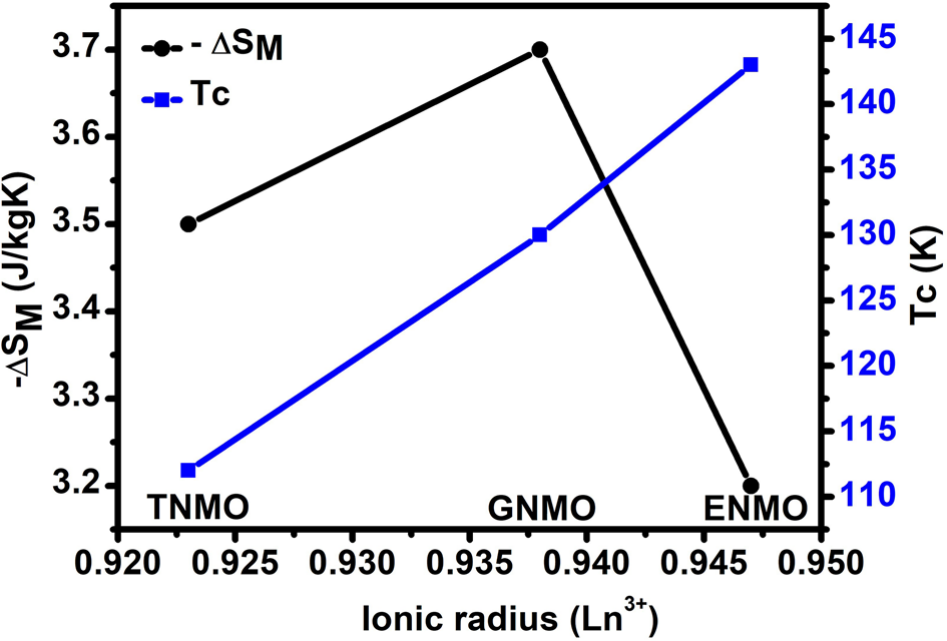
Magnetic entropy changes and Curie temperature of ENMO, GNMO, and TNMO samples with respect to ionic radius. With increasing ionic radius of rare earth element Eu, Gd, and Tb, Curie temperature increases while – ΔS_M_ does show significant change.

**Figure 7:**
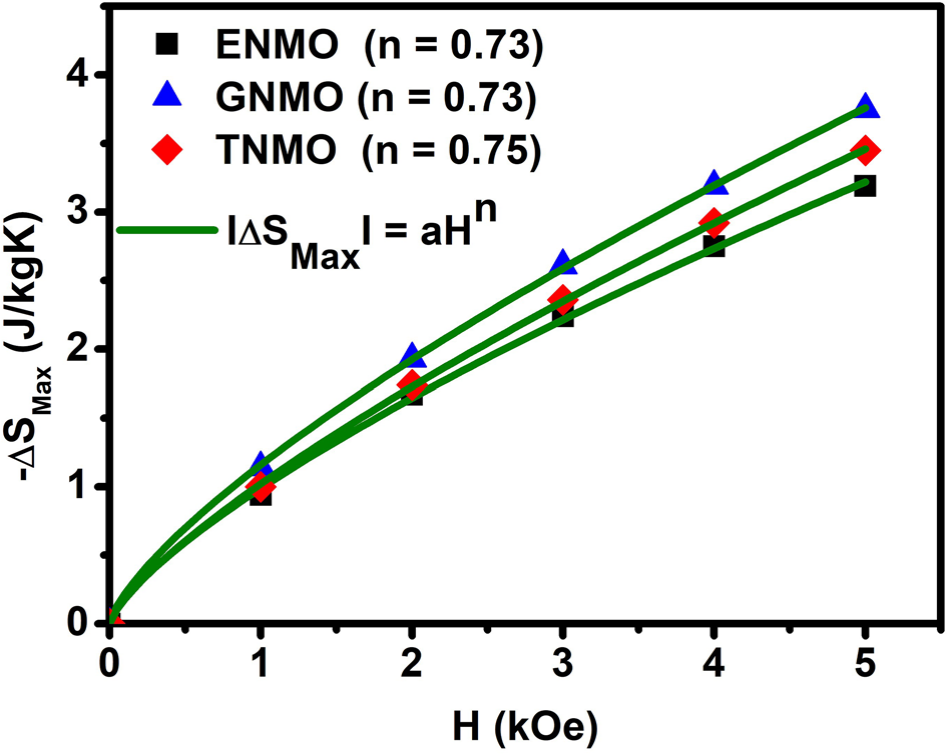
|ΔS_Max_| data of ENMO, GNMO, and TNMO samples fitted to a function |△S_Max_|= α × H^n^, where the values of exponent “n” are labelled in the figure.

The calculated RCP values for ENMO, GNMO, and TNMO samples are 150, 182, and 176 J/kg, respectively. Table 2 summarizes the comparison of transition temperature, magnetic entropy change, and RCP values for the investigated samples and other reported double perovskite compounds. The – ΔS_M_ and RCP values for ENMO, GNMO, and TNMO does not show much change with different rare earth elements in double perovskite, however there is shift in Curie temperature (T_C_) observed. Table 2 show that the MCE characteristics for the investigated DPs samples are comparable with other DPs materials, showing that ENMO, GNMO, and TNMO samples are also important for magnetic cooling applications. The comparative research given in the table, the Curie temperature falls from 143 to 84 K with decreasing ionic radii of the rare earth element in Ln_2_NiMnO_6_ (Ln = Eu, Gd, Tb, Dy, Ho, and Er) double perovskite, however there are no significant changes in MCE and RCP values. Figure 6 depicts the magnetic entropy change and Curie temperature of ENMO, GNMO, and TNMO samples with respect to increasing ionic radius of rare earth elements (Eu, Gd, and Tb). The magnetic entropy change with different A-site element of the investigated double perovskite varies and does not display a simple monotonic trend as the Curie temperature falls gradually. To determine the order of magnetic phase transition another technique was described in the literature, in which the field dependence of − ΔS_M_ of the sample was determined by utilizing the relation ΔS_Max_ = aH^n^, where a is constant and n is an exponent linked to the magnetic order^40,41^. Figure 7 depicts − ΔS_Max_ with respect to magnetic field along with power law fitting, and the resulting values of “n” are 0.73, 0.73, and 0.75 for ENMO, GNMO, and TNMO samples, which are somewhat higher than mean field ferromagnets (n = 0.67). However, for single phase ferromagnets, the exponent “n” is considered as function of magnetic field and temperature^2,42^, and is written as,

4 $${\text{N }}\left( {{\text{T}},{\text{ H}}} \right) \, = \frac{{dln\left| {{ }\Delta S_{M } \left( {{\text{T}},{\text{ H}}} \right){ }} \right|}}{dln\left( H \right)}$$

**Table 2:** Comparison of – ΔS_M_ and RCP values for the R_2_NiMnO_6_ (R = Eu, Gd, Tb, Dy, Ho, Er) samples and other double perovskite compounds with different A-site and B-site elements.

Compound	Tc (K)	ΔH (T)	– ΔS_M_ (J/kg K)	RCP (J/kg)	Ref.
Eu_2_NiMnO_6_	143	5	3.2	150	Present work
Gd_2_NiMnO_6_	130	5	3.7	182	Present work
Tb_2_NiMnO_6_	112	5	3.5	176	Present work
Dy_2_NiMnO_6_	101	5	3.4	175	^20^
Ho_2_NiMnO_6_	93	5	3.7	194	^20^
Er_2_NiMnO_6_	84	5	3.4	169	^20^
Pr_2_CoMnO_6_	176	5	2.1	–-	^36^
Eu_2_CoMnO_6_	123	6	3.3	–-	^37^
Dy_2_CoMnO_6_	8.5	5	8.7	170	^38^
Er_2_CoMnO_6_	7.5	5	9.2	148	^38^
La_2_CrMnO_6_	112	7	1.31	138	^39^
Pr_2_CrMnO_6_	93	7	1.86	173	^39^
Nd_2_CrMnO_6_	80	7	2.23	190	^39^

**Figure 8:**
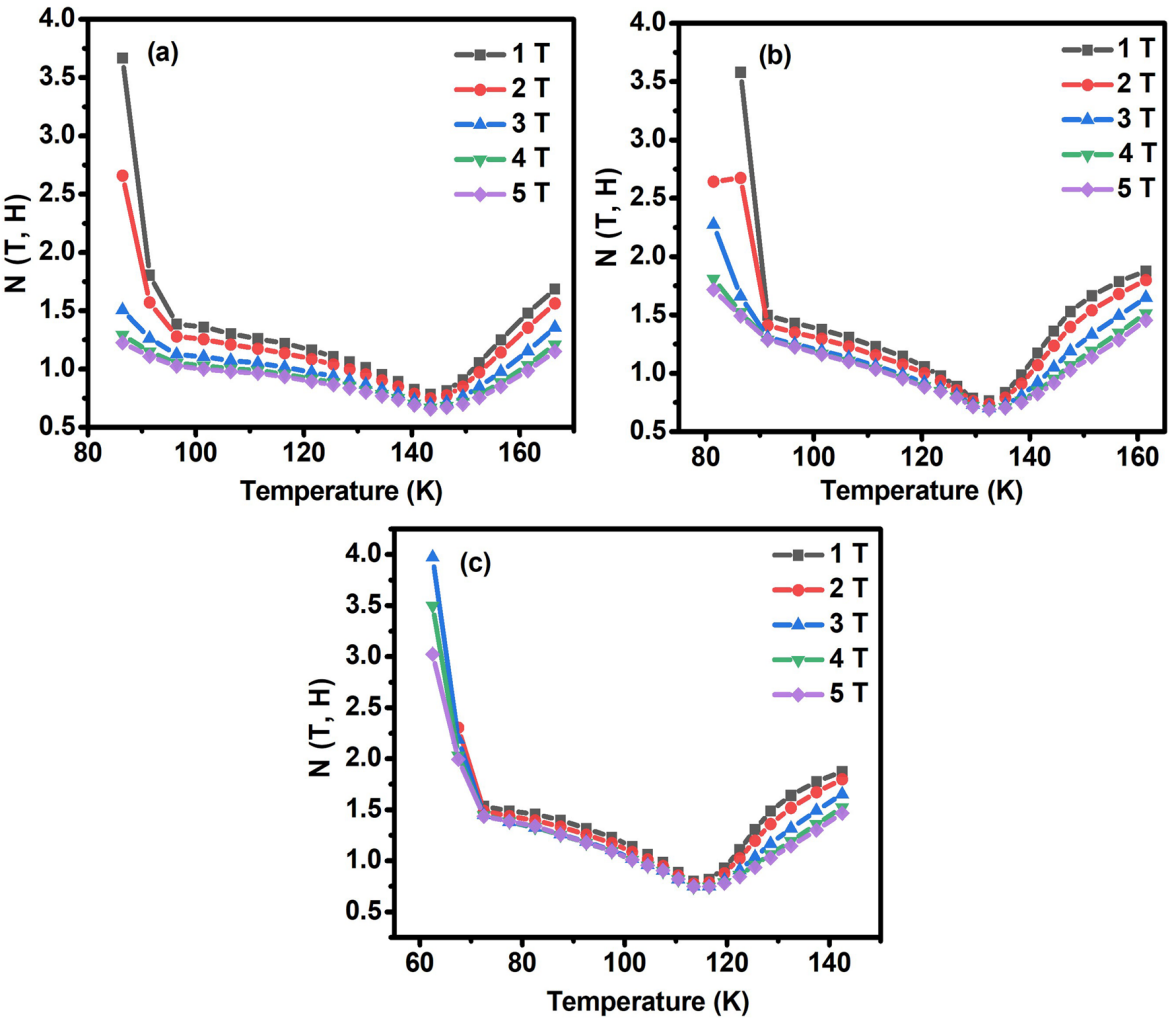
Temperature dependence of the exponent N (T, H) at various magnetic field for the (**a**) ENMO, (**b**) GNMO, (**c**) TNMO samples at different magnetic field.

The magnetic order of ferromagnets is characterized by the values of N (T, H) and n. According to mean-field theory, N (T, H) at T_C_ is independent of H and achieves the value, with n = N (T, H) = 2/3, for the ferromagnet accompanied by long-range magnetic order, whereas it tends to 1 and 2 as T ≪ T_C_ and T ≫ T_C_, respectively. The calculated N (T, H) data using the |ΔS_M_ (T, H)| data shown in the magnetic entropy change curve, as shown in Fig. 8a–c, indicating that N (T, H) is significantly dependent on temperature and magnetic field. The values of N (T, H) at T = Tc for ENMO, GNMO, and TNMO samples are 0.66, 0.69, and 0.75, respectively. In ferromagnetic region, T ≪ T_C_, exponent, N values increase up to value 4, but tend to settle around value 1. At T_C_, the N (T, H) values are very close to the n values obtained by fitting the |ΔS_M_ (H)| data to the power function |ΔS_M_ (H)|∝ H^n^. The obtained values of n and N (T, H) at T = Tc imply that all the investigated samples experience second-order magnetic phase transition. The difference in N (T, H) and n values in the ferromagnetic state as well as N (T, H) is seen to be more than 2, demonstrating that the presence of Mn^3+^ and Mn^4+^ ions induces ferromagnetic and antiferromagnetic interactions in double perovskite samples. However, values of N (T, H) greater than 2 indicate the presence of a first-order magnetic phase transition at lower temperatures^43^. Expecting a change in MCE properties with different rare earth elements having different ionic radii in *A*_*2*_*BB’O*_*6*_ double perovskite is not much feasible, because of phase transition in double perovskite is associated with M^2+^–O–Mn^4+^ superexchange interaction but playing with working temperature this could be one of tool to tune the Curie temperature.

## Conclusions

In summary, we used solid-state method to synthesize Eu_2_NiMnO_6_ (ENMO), Gd_2_NiMnO_6_ (GNMO) and Tb_2_NiMnO_6_ (TNMO) double perovskite, and we discovered that all the samples have monoclinic structure with P2_1_/n space group. XRD, XPS, MT, and MCE data have been systematically investigated. All the samples exhibit a second-order magnetic phase transition, with curie temperatures (T_C_) of 143, 130, and 112 K for ENMO, GNMO, and TNMO, respectively. The drop in Curie temperature is owing to a decrease in the ionic radii of Eu, Gd, and Tb in the double perovskite structure, which is related with the structural disorder and superexchange interaction. The – ΔS_M_ and RCP at an applied field of 5 T are found to be 3.2, 3.8 J/kg K, 3.5 J/kg K and 150, 182, 176 J/kg K respectively, for the studied samples. The – ΔS_M_ and RCP suggest that the investigated compounds Eu_2_NiMnO_6_, Gd_2_NiMnO_6_, and Tb_2_NiMnO_6_ may be considered as magnetic refrigerants with wider temperature range and making them potential magnetic refrigerant materials.

## Experimental

Polycrystalline double perovskite compounds of Eu_2_NiMnO_6_ (ENMO), Gd_2_NiMnO_6_ (GNMO), Tb_2_NiMnO_6_ (TNMO) in the present study were synthesized by conventional solid-state method. The stoichiometric amount of the precursors Eu_2_O_3_, Gd_2_O_3_, Tb_2_O_3_, NiO, and MnO_2_ were combined and ground in the mortar before being heat-treated at 900 °C for 24 h, after regrinding heat-treated at 1100 °C for 24 h, and finally all samples sintered at 1300 °C for 48 h after regrinding. The phase formation of sintered ceramic compounds was investigated by X-ray diffraction (XRD) by using the X-ray diffractometer (Rigaku) and analyzed by Rietveld method by using TOPAS software. The X-ray photoelectron spectroscopy (XPS, VersaProbe, PHI 5000) experiment was performed using an Al-K_α_ radiation source under 15 kV voltage and 5 mA current. The XPS data was examined by using the XPSpeak41 software. The temperature dependent magnetization and isothermal magnetization of the samples were determined using a vibrating sample magnetometer (VSM) based on Superconducting Quantum Interference Device (SQUID) from Quantum Design.
